# Prenatal chlorpyrifos exposure alters motor behavior and ultrasonic vocalization in cd-1 mouse pups

**DOI:** 10.1186/1476-069X-8-12

**Published:** 2009-03-30

**Authors:** Aldina Venerosi, Laura Ricceri, Maria Luisa Scattoni, Gemma Calamandrei

**Affiliations:** 1 Section of Neurotoxicology and Neuroendocrinology Department of Cell Biology and Neuroscience, Istituto Superiore di Sanità, Viale Regina Elena 299, I-00161 Roma, Italy

## Abstract

**Background:**

Chlorpyrifos (CPF) is a non-persistent organophosphate (OP) largely used as pesticide. Studies from animal models indicate that CPF is a developmental neurotoxicant able to target immature central nervous system at dose levels well below the threshold of systemic toxicity. So far, few data are available on the potential short- and long-term adverse effects in children deriving from low-level exposures during prenatal life and infancy.

**Methods:**

Late gestational exposure [gestational day (GD) 14–17] to CPF at the dose of 6 mg/kg was evaluated in CD-1 mice during early development, by assessment of somatic and sensorimotor maturation [reflex-battery on postnatal days (PNDs) 3, 6, 9, 12 and 15] and ultrasound emission after isolation from the mother and siblings (PNDs 4, 7 and 10). Pups' motor skills were assessed in a spontaneous activity test on PND 12. Maternal behavior of lactating dams in the home cage and in response to presentation of a pup previously removed from the nest was scored on PND 4, to verify potential alterations in maternal care directly induced by CPF administration.

**Results:**

As for the effects on the offspring, results indicated that on PND 10, CPF significantly decreased number and duration of ultrasonic calls while increasing latency to emit the first call after isolation. Prenatal CPF also reduced motor behavior on PND 12, while a tendency to hyporeflexia was observed in CPF pups by means of reflex-battery scoring. Dams administered during gestation with CPF showed baseline levels of maternal care comparable to those of controls, but higher levels of both pup-directed (licking) and explorative (wall rearing) responses.

**Conclusion:**

Overall our results are consistent with previous epidemiological data on OP neurobehavioral toxicity, and also indicate ultrasonic vocalization as an early marker of CPF exposure during development in rodent studies, with potential translational value to human infants.

## Background

The OP chlorpyrifos is a non-persistent insecticide widely employed in domestic, agricultural and non-agricultural (i.e. schools, golf courses, parks) settings. Its toxicity, related to inhibition of brain and systemic acetylcholinesterase (AChE), is well documented after acute poisoning of adults. The evaluation of CPF neurotoxicity after sub-toxic exposure and in developing organisms appears more controversial, as most of available animal studies indicates that CPF exposure below the threshold for systemic toxicity exerts disruptive effects on CNS development and behavior [1-12]. In the last decade, increased concern has been raised about adverse effects of pesticides on central nervous system (CNS) development [13,14]. Prolonged exposure, multiple ways of exposure, and exposure to mixture of pesticides could indeed determine – also at apparently sub-toxic doses – a level of CPF burden compatible with increased health risk. The US Environmental Protection Agency (EPA) imposed a ban on its sale for residential use [15], thus the use of CPF in the USA has been restricted to agricultural applications only. However, agricultural and non-agricultural use remains of some concern and the final report of Interim Reregistration Eligibility Decision foresees mitigation measures to reduce some occupational and ecological exposures by eliminating use sites and reducing application rates [16]. In Europe, regardless of the wide and frequently OPs use, with CPF the top selling insecticide [17], no restrictions of use site or application rate are currently required [18].

A recent review [19] summarizes epidemiological studies that support the developmental neurotoxicity of OPs, although limitations of the available data were overtly admitted. In the CHAMACOS cohort study, including women resident in an area of major agricultural production, the presence of the OP metabolite dialkylphosphate (DAP) in maternal urine or blood was associated with impaired reflex functioning in infants after PND 3 [20]. Similar data are reported in a birth cohort study from New York City [21]. Impairment in mental and psychomotor performance and attention problems in infants assessed at 12, 24, and 36 months were found to be associated with CPF levels in the cord blood in a longitudinal birth study of inner-city mothers [22]. Comparable behavioral problems were reported in the CHAMACOS cohort in 24-month-old children [23].

Despite results from epidemiological studies indicate that some effects of developmental exposure to CPF are already evident in early infancy, few rodent studies so far have focused on the behavioral effects of CPF in the early developmental phases. In preweaning rats righting reflex and cliff avoidance tests were markedly altered following repeated, low-level CPF exposures during late gestation [24]. Deficits in righting reflex and geotaxis response were also reported in rat female pups after PND 1–4 exposure [6]. In a mouse model of gene-environment interactions, prenatal chlorpyrifos exposure per se induced an accelerating effect on maturation of grasping reflex in mutant Reeler mice [25].

Altricial species, such as rodents, may represent a useful animal model to mimic the immature development of body and motor skills in humans at birth [26]. In rodents several reflexes and behavioral responses show a remarkable regularity in their time of appearance and subsequent maturation, thus representing a reliable tool for assessing abnormalities in early neurodevelopment [27,28]. Batteries of developmental milestones have been designed to describe early neurodevelopment of newborn rodents and include behavioral markers of maturation of proprioception (tactile response such as grasping, placing etc), and vestibular function which involves acquisition of coordination and adequate strength [29,30]. The ontogenetic profile of rodent neurobehavioral development can also be measured by the analysis of the species-specific emission of ultrasound vocalizations (USVs) which are characterized by frequencies ranging from 30 to 90 kHz. USVs present a clear ontogenetic profile peaking around day eight after birth and decreasing close to zero when pups are 2-weeks old [31-33], and are elicited in neonate rodents by several environmental and pharmacological stimuli [34,35]. Several experimental evidences support USVs as indicators of the emotional state of neonate rodents [36,37] with an important role in the establishment of the mother – offspring bond [38].

In this study we evaluate the effects of late gestational CPF exposure (GD 14–17) on early neurobehavioral development in mouse pups in order to extend our previous studies on CPF developmental exposure, that were principally focused on long-term neurobehavioral effects. The dose of CPF selected (6 mg/kg) does not elicit systemic toxicity in pregnant females and their offspring and fails to inhibit brain AChE of pups at birth as previously shown using the same CPF dose, and this same treatment schedule in the CD-1 mouse strain [10]. Throughout the first fifteen postnatal days, analysis of sensorimotor reflex maturation was carried out, and the ontogenetic profile of USV emission after isolation from the mother was assessed on PND 4, 7, and 10. Our interest towards USV emission was threefold: 1) USV are one of the few early markers of neurobehavioral development in rodent models; 2) we found in our previous studies on developmental CPF exposure long-term alterations in behaviors that reflect emotional/affective states in mice, such as agonistic behavior in adult males [10], maternal aggression [12], social novelty [11] and maternal behavior [10,12], and USV are an appropriate end point to assess emotional responses early in life; 3) developmental CPF exposure is reported to interfere with the expression of serotonergic receptors as well as with serotonin turn-over [2,8], and to increase oxytocin hypothalamic levels [39]. Ultrasonic calls are modulated both by serotonergic and oxytocinergic neurotransmission [40-43]. We also analyzed spontaneous behavior of pups on PND 12, to evaluate overall level of activity and quality of the age-specific motility repertoire. In addition, in order to evidence potential changes in females' behavior induced by gestational CPF administration, a selection of maternal responses of lactating females was assessed when offspring were 4-days old.

## Methods

### Animals and Treatments

All experiments on animals were performed according to the European Community Council Directive 86/609/EEC and to Italian Legislation on Animal Experimentation (Legislative Decree 116/92). Male and female mice of a Swiss-derived outbreed strain (CD-1, Harlan, S. Pietro al Natisone, Italy), were housed in breeding cages with a 12-hr light-dark cycle (light on 20:00-8:00) and with free access to food and water. Females were inspected daily for the presence of the vaginal plug (GD 0). The stud was removed 10 days after the discovery of the vaginal plug. On GD 15 forty females were randomly assigned to one of the two prenatal treatments [vehicle (Veh), CPF]. CPF (Chem. Service, West Chester, PA) was dissolved in peanut oil (Veh) to provide rapid and complete absorption. CPF (in a volume of 0.1 ml/10 g at a dose of 6 mg/kg) or its vehicle was administered to pregnant females from GD 15 to 18 by intraoral gavages. We have previously shown a 75% serum AChE inhibition 24 hr after termination of same CPF treatment in pregnant females, while brain AChE activity was reduced to 60% of control values, an effect no longer detectable 24 h later [10]. However, the observed AChE inhibition was not associated with any sign of systemic toxicity and/or weight loss in pregnant females or altered reproductive performances (fetus viability, number of pups delivered, sex ratio and mean pups' weight at birth). Furthermore, this CPF treatment schedule did not affect brain AChE activity in offspring, but causes a mild transient inhibition (20% of control values) in serum AChE activity at birth [10].

Thirty-four litters (18 Vehicle-treated and 16 CPF-treated) were used and culled at birth to five males and five females. Assessment of sex of the pups was done by evaluation of anogenital distance [44]. On the day of birth, three pups were tattooed with permanent ink on their limbs for individual identification and randomly assigned to one of the following experiment: sensorimotor assessment and spontaneous behavior (one male and one female from each litter), USV recording (one male). The remaining pups were left undisturbed till weaning and subsequently assigned to a different experiment.

### Dams' behavior

A selection of maternal responses expressed by individual dams (Veh, n = 15; CPF, n = 15) was recorded on post-partum day 4. All observations (about 10 minutes each) were made under red light in an experimental room separated from the animal colony and kept in standard environmental conditions. The observations were made between 10:00 and 14:00 hr and were performed by a trained observer blind to the assignment of the dams to the different treatment groups. Each observation consisted of two sessions: Session 1 (8 min): after removal of one male pup [this pup was concomitantly evaluated for USV in a sound proof experimental room (see below)]. Session 2 (2 min): after this same male pup was returned to the home cage.

The following behavioral categories were considered: Pup-directed behaviors – Retrieving: the female is picking up the pup in her mouth and carrying it to the nest; Licking: the animal is licking any part of the pup's body, primarily the anon-genital region; Sniffing: the female is sniffing one or more pups; Crouching: the female's body is arched over the pups with no other apparent movement. Non pup-directed behaviors – Digging: digging in the sawdust out of nest side, pushing and kicking it around using the snout and/or both forepaws and hind paws, in most cases the animals are moving around the cage, sometimes changing the whole arrangement of the substrate material; Self-grooming: the female is wiping, licking, combing or scratching any part of its own body; Wall Rearing: the female rears on hind limbs, while leaning with the forelimbs on the cage walls (or not), often sniffing the air; Locomotion: body movements assessed by number of crossing of two virtual lines dividing the cage in three sectors on the screen during videotape analysis; Resting.

Frequency and duration of each behavioral category were collected using The Observer software (Noldus, Wageningen, NL) by an individual blind to the experimental conditions.

### Assessment of Somatic and Behavioral Development (PNDs 3, 6, 9, 12 and 15)

On PND 3, 6, 9, 12, and 15, one male and one female from each litter in each treatment group (Veh n = 18 M, 18 F; CPF n = 16 M, 16 F) were assessed for somatic and neurobehavioral development.

Pups were weighed to the nearest 0.01 g and their body was measured by a flexible rule to the nearest 0.1 cm. Hair growth, day of eyelid opening, and incisor eruption were also recorded.

Pups were then assessed for a number of measures currently used in the study of sensorimotor ontogeny in mice according to a slightly modified Fox battery [25,29,45]. The tests were conducted during the dark period between 09:00 and 14:00 hr under red light, each subject being tested at approximately the same time of the day. The following reflexes and responses were considered:

#### Quantitative data

Righting reflex: pup turns over with all four feet on the ground when placed on its back. The reflex was tested three times with a cut-off latency of 30 s. Data for the best performance (shorter latency) and data for the worst performance (longer latency) were analyzed separately; Grasping reflex: to evaluate the strength of grasping reflex, pups were placed on a narrow mesh metal grid in an horizontal position. The grid was progressively tilted to a vertical position (+ 90°) and further to a horizontal upside-down position (+ 180°), the angle of the grid at which pup falls is termed "fall angle". The test was repeated thrice, and the mean angle reached was recorded; Cliff aversion: pup withdraws from the edge of a flat surface when its snout and forepaws are placed over the "cliff", time of aversion was recorded with a cut-off of 10 s.

#### Qualitative data

Forelimb and hind limb placing reflexes: pup raises and places its fore- or hind paw on the surface of the edge of an object when stroked on the dorsum of the paw; Forelimb and hind limb stick grasp reflexes: pup grasps a toothpick when the fore- or hind paw is stroked; Pole grasping: pup grips a wooden pencil with its forepaws; The following scores were used for qualitative somatic and behavioral variables so far mentioned: 0 = no response; 1 = uncertain response; 2 = incomplete response; 3 = full response.

### Ultrasonic Vocalization (PNDs 4, 7, 10)

Ultrasonic calls of one male from each litter in each treatment group (Veh n = 15; CPF n = 15) were recorded in a sound-attenuating chamber (Amplisilence, I-10070 Robassomero, Italy) during the dark period between 10:00 and 14:00 hr. Single pups were removed from the home cage and individually placed in a glass container (diameter 5 cm, height 10 cm). The number of ultrasonic calls emitted during the 4 min test was assessed using an ultrasonic microphone (Avisoft UltraSoundGate condenser microphone capsule CM16, Avisoft Bioacoustics, Berlin, Germany) sensitive to frequencies between 10–180 kHz was suspended 10 cm above the glass. Vocalizations were recorded using an Avisoft Recorder (Version 3.2). Settings included sampling rate at 250 kHz; format 16 bit. For acoustical analysis, recordings were transferred to Avisoft SASLab Pro (Version 4.40) and a fast Fourier transformation (FFT) was conducted. Spectrograms were generated with an FFT-length of 512 points and a time window overlap of 75% (100% Frame, Hamming window). The spectrogram was produced at a frequency resolution of 488 Hz and a time resolution of 1 ms. A lower cut-off frequency of 15 kHz was used to reduce background noise outside the relevant frequency band to 0 dB. Call detection was provided by an automatic threshold-based algorithm and a hold-time mechanism (hold time: 0.005 s). An experienced user checked the accuracy of call detection, and obtained a 100% concordance between automated and observational detection. Parameters analyzed included for each test day number of calls, duration of calls, frequency (kHz) and amplitude at maximum of the spectrum.

At the end of the 4 min recording session, the axillary temperature of each pup was measured by gentle insertion of the thermal probe in the skin pocket between upper foreleg and chest of the animal for about 30 s (Microprobe digital thermometer with mouse probe, Stoelting Co., Illinois, USA).

### Spontaneous motor behavior (PND 12)

One male and one female pup from each litter assigned to the two experimental conditions were assessed for their spontaneous motor behavior on PND 12 (Veh, n = 13 M, 13 F; CPF, n = 14 M, 14 F). All observations (one session of 5 min) were made under red light in an experimental room separated from the animal colony and kept in standard environmental conditions. The observations were made between 10:00 and 14:00 hr. Pups were individually introduced in a glass cylinder (14 cm diameter, 8 cm height) with adsorbent paper on the floor kept in an incubator set at 30 ± 1°C.

The following behavioral items were collected: Crossing (forward movements of the body); Immobility (no visible movements of the body); Head moving (head raising and head turning); Wall climbing; Pivoting (turning to left or right propelled only by forelimbs; hind limbs stationary); Grooming (signs of wiping).

Frequency and duration of each behavioral category were collected using The Observer software (Noldus, Wageningen, NL) by an experimenter blind to the experimental condition of the animals.

### Statistical analysis

Parametric analysis of variance (ANOVA) was performed on frequency and duration of dams' behavior responses, USV data, pups' body weight and length, Grasping, Righting and Cliff avoidance data. Specifically: ANOVA model for maternal behavior data included prenatal treatment (2 levels) as between-subject fixed factor; ANOVA model for USV data included prenatal treatment (2 levels) as between-subject fixed factor and days as repeated measure factor (3 levels); ANOVA model for pups' body weight and length, Grasping, Righting and Cliff avoidance reflexes included prenatal treatment (2 levels) as between-litter fixed factor, litter as random blocking factor nested within prenatal treatment and blocking factor for sex and days, sex as within-litter fixed factor (2 levels) and day as repeated measure factor (5 levels).

Posthoc comparisons were performed using Tukey's HSD test, which can be used in the absence of significant ANOVA results [46].

Non-parametric analyses of variance (ANOVAs) were performed on qualitative data for sensorimotor maturation: for each reflex a synthetic measure of the score profile across days was computed (area under the curve generated by the repeated scores) and Mann-Whitney U test was applied. Moreover, in order to obtain information on differences in maturation level across days, a Total Daily Score was computed for each subject in each day of testing, by summing the scores of all reflexes recorded on that subject in that day. The Total Daily Score was analyzed by parametric ANOVA, including prenatal treatment (2 levels) as between-litter fixed factor, litter as random blocking factor nested within prenatal treatment and blocking factor for sex and days, sex as within-litter fixed factor (2 levels) and day as repeated measures factor (5 levels). The Mann-Whitney U test was also performed day-by-day. When performing the Mann-Whitney U test (for both area under the curve and Total Daily Score), the response variable was transformed by summing the observations on the two subjects (one male and one female) per litter, to assess main effect of prenatal treatment, and by computing the difference between the observations from the same two subjects, to assess the interaction prenatal treatment x sex. Data are reported by means ± SEM.

## Results

### Reproductive performance

On PND 0 data analysis on number of delivered pups (Veh: 11.5 ± 0.5 CPF: 10.2 ± 0.7); sex rate (% males Veh: 41.6 ± 2.6, CPF: 42.3 ± 3.6; % females Veh: 57.0 ± 2.8, CPF: 51.8 ± 4.1) and overall litter weight (Veh, 20.19 ± 0.91 g; CPF, 19.81 ± 1.15 g) did not show any detrimental effect of gestational CPF exposure.

### Dams' behavior

CPF gestational exposure did not affect maternal responses recorded during Session 1 (data not shown).

**Figure 1:**
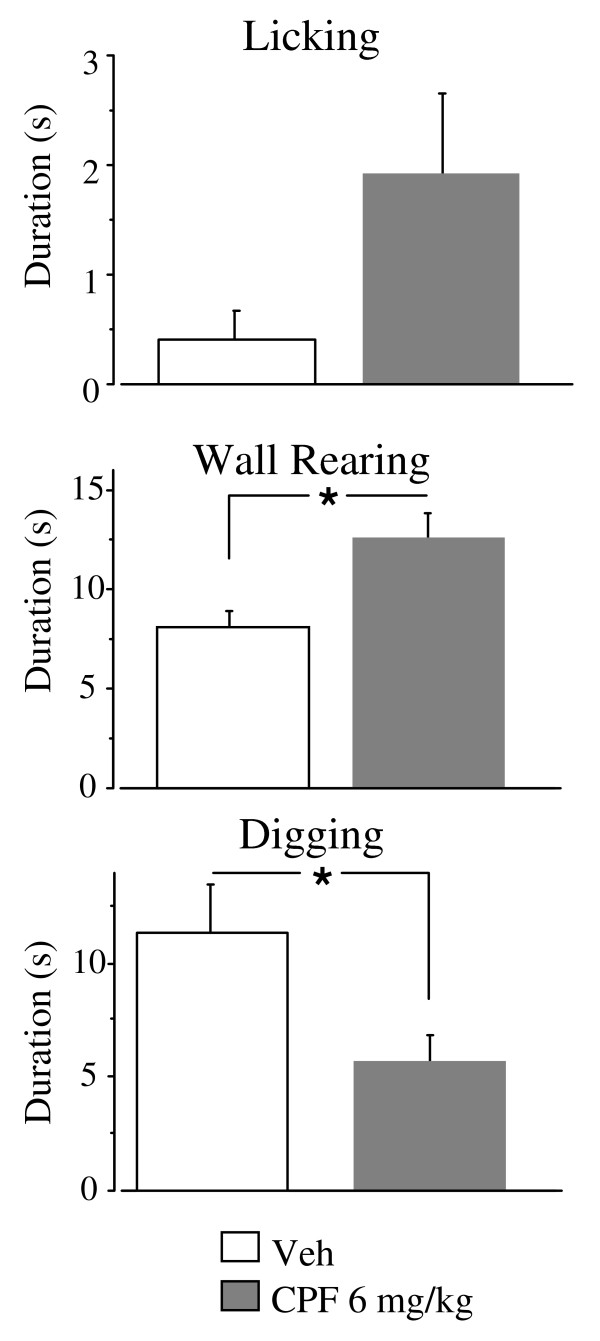
**Dams' Behavior**. Selected pup-directed and non pup-directed behaviors displayed by Veh and CPF treated females on postpartum day 4, after the pup was returned to the home cage. Session lasted two minutes. * p < 0.05. Veh, n = 15; CPF, n = 15.

During Session 2, latency to retrieve the male pup returned to the home cage was not altered by CPF. As for Licking response towards all the pups, CPF tended to increase its duration [F (1, 28) = 3.44 p = 0.07] (see Figure 1). The other pup-directed maternal behaviors were not affected by CPF during Session 2. As for non pup-directed behaviors, CPF significantly increased Wall rearing [duration: F (1, 28) = 7.85 p = 0.01; frequency: F (1, 28) = 3.99 p = 0.05] and decreased Digging duration [F (1, 28) = 6.46 p = 0.01].

### Assessment of Somatic and Neurobehavioral Development (PNDs 3, 6, 9, 12, and 15)

A significant treatment x sex interaction was found for Body length [F (1, 32) = 8.67 p < 0.01], with CPF males being shorter than Veh controls (post hoc comparisons p < 0.05, see Table 1). As for body weight gain neither a main effect of CPF nor a CPF interaction with treatment, sex or day was found.

**Table 1:** Somatic and reflex maturation of pups prenatally exposed to Veh or CPF (6 mg/kg).

		Body Lenght (cm)				
		3	6	9	12	15
Veh	m	3.04 ± 0.03	3.34 ± 0.03	3.67 ± 0.04	4.06 ± 0.07	4.54 ± 0.08
CPF *		2.97 ± 0.05	3.27 ± 0.04	3.61 ± 0.05	3.86 ± 0.06	4.29 ± 0.08
						
Veh	f	3.02 ± 0.04	3.36 ± 0.04	3.61 ± 0.04	4.04 ± 0.06	4.43 ± 0.08
CPF		3.00 ± 0.04	3.38 ± 0.04	3.64 ± 0.04	4.02 ± 0.07	4.44 ± 0.09

		Body Weight (g)				

		3	6	9	12	15
Veh	m	2.78 ± 0.06	4.61 ± 0.08	6.51 ± 0.18	7.84 ± 0.23	9.12 ± 0.25
CPF		2.88 ± 0.06	4.67 ± 0.12	6.45 ± 0.19	7.63 ± 0.22	8.94 ± 0.22
						
Veh	f	2.77 ± 0.06	4.39 ± 0.13	6.14 ± 0.18	7.74 ± 0.22	9.06 ± 0.23
CPF		2.77 ± 0.09	4.57 ± 0.12	6.39 ± 0.12	7.71 ± 0.18	8.98 ± 0.20

		Total daily qualitative score				

		3	6	9	12	15
Veh		2.78 ± 0.17	5.28 ± 0.16	8.66 ± 0.18	12.44 ± 0.22	18.44 ± 0.22
CPF		2.53 ± 0.12	5.09 ± 0.14	8.69 ± 0.15	11.97 ± 0.24	17.50 ± 0.29

Data are indicated as (Mean ± SEM).

* p < 0.05 after posthoc comparisons, see Results

As for quantitative data on sensorimotor coordination development, CPF did not affect Grasping, Righting and Cliff aversion reflexes.

Non-parametric analysis conducted on each reflex score profile across days only evidenced a trend towards delayed appearance of Hind limb grasping in CPF exposed pups [Mann-Whitney U (18,16) = 90.5 p = 0.06]. As for the Total Daily Score, sum of the single qualitative daily reflex scores, parametric analysis showed a main effect of treatment (mean data shown in Table 1) just missing statistical significance [F (1, 30) = 3.65 p = 0.06] and no interaction of treatment with sex or day; the non-parametric analysis, conducted on the Total Daily Score in each day, evidenced a lower score in CPF exposed pups on PND15 [U (16, 16) = 79 p = 0.06].

### Ultrasonic Vocalization (PNDs 4, 7, 10)

**Figure 2:**
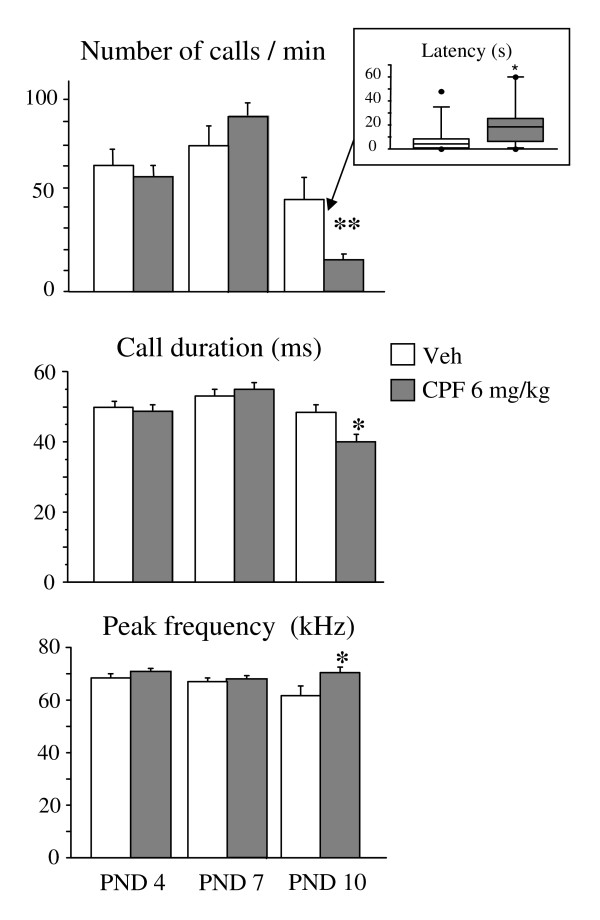
**Ultrasound vocalization parameters**. Quantitative ultrasound vocalization data recorded on PND 4, 7, and 10 during a 4- min session. * p < 0.05, ** p < 0.01. Inset graph (upper panel): Latency to emit the first call on PND10, data are median ± interquartile range. Veh n = 15 (only males); CPF, n = 15 (only males).

A significant interaction between CPF and day was found for number and duration of ultrasonic calls [F (1, 56) = 4.65 p = 0.01; F (1, 56) = 3.56 p = 0.03 respectively]. Post hoc comparisons showed that CPF decreased both number and duration of calls of CPF male pups on PND 10 (Ps < 0.05). Peak frequency of calls was significantly increased in CPF pups on PND 10 [treatment x day interaction: F (2, 56) = 2.41 p = 0.09, p < 0.05 after post hoc comparisons], while no effect was observed for Peak amplitude. Finally, a significant CPF delaying effect was found for the emission of the first call [U (15, 15) = 65 p = 0.04] on PND 10 (see Figure 2).

### Spontaneous behavior (PND 12)

**Figure 3:**
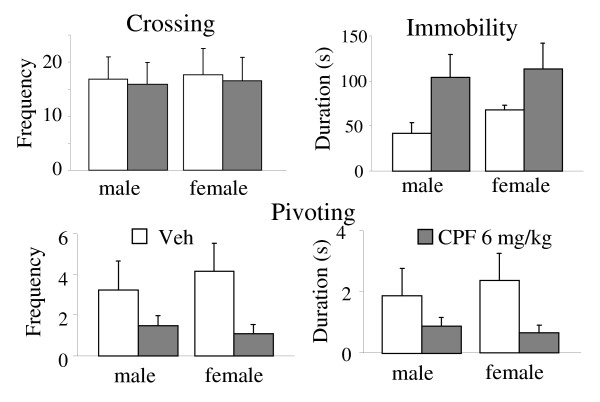
**Spontaneous behavior of 12-day-old pups**. Behavioral responses recorded during the Spontaneous behavior test on PND 12. As for Pivoting and Immobility behavior a main effect of the treatment was found (p < 0.05; see Results section). Veh, n= 13 (both males and females); CPF, n = 14 (both males and females).

CPF did not interfere with the overall level of locomotor activity measured by number of crossings. However, CPF pups displayed significantly less Pivoting behavior [F (1, 25) = 5.82 p = 0.02, F (1, 25) = 5.19 p = 0.03 for frequency and duration respectively,] than control pups and showed a parallel increase of Immobility [F (1, 25) = 4.28 p = 0.05, F (1, 25) = 4.67 p = 0.04 for frequency and duration respectively] (see Figure 3).

## Discussion

Overall the results of the present study show that administration of CPF in late gestation results in early behavioral alterations in mouse pups. The assessment of neurodevelopment across the first 15 days after birth indicates a significant depressive effect of CPF on pup behavior after day 10th of postnatal life that concerns both distress response to isolation from the mother and motor skills.

The analysis of somatic development indicates that CPF failed to affect body weight but reduced body length in male pups. This effect, that is suggestive of a subtle growth delay, is in partial agreement with what reported by one epidemiological study [47] evidencing decreased somatic growth at birth in neonates born from urban minorities women in New York City, associated with cord plasma CPF levels.

As for sensorimotor development, only a general trend towards hyporeflexia was shown by CPF mice when considering either a total score of maturation or single reflex analysis (hind limb grasping). Indeed, all effects on reflexes remain at the margin of significance and thus lying in the uncomfortable range where definitive conclusions about a retarding CPF effect cannot be drawn. In the rat species perinatal CPF administration at a dose comparable with the one used in the present study was found to delay early somatic and sensorimotor development of the offspring [24,48]. However in these two rat studies CPF was administered for longer periods than in the present mouse study. Thus it is possible that only when CPF exposure covers a large portion of gestation it induces statistically detectable neurodevelopmental delays. In humans too abnormal reflexes have been reported after prolonged exposure, namely either in infants born from mothers exposed to different OPs from a urban cohort [21], and from a Californian cohort of farm working women [20].

The clear depressive effect of prenatal CPF on ultrasonic vocalizations observed in 10-day-old pups represents the main finding of the present study. CPF exposure resulted in lower reactivity to isolation (higher latency to emit the first call), decreased number and duration of vocalizations, and finally in a higher peak frequency of the calls. Furthermore, the altered profile of ultrasound emission was accompanied by significant changes in spontaneous motor behavior on PND 12: CPF-exposed pups displayed less Pivoting – a typical neonatal motor pattern in rodents – associated with a higher level of immobility. It is worth noting that a selective impairment in early motor reflexes, such as crawling, has been observed in newborns exposed prenatally to OPs [20,21].

A large body of data define USV emission as a phenomenon that may contribute both to maintenance of stable body temperature and homeostasis [49], playing at the same time a communicative role in stimulating the mother to look for and retrieve the pup, thus functioning as an indicator of the affective state of the pup [50]. In a translational perspective, it is remarkable that rather similar hypotheses have been drawn to interpret human infant cry. Organs and structures (larynx, thoracic and abdominal muscles etc.) and neurological systems which modulate infant vocalizations (vagal nerve, brainstem and limbic system) are widely shared in mammals [34,51,52]. A large number of studies on rodent models clearly demonstrate that USVs are a sensitive (and almost unique) and reliable marker to detect short-term neurotoxic effects of developmental exposure to environmental contaminants [53-55]. USVs are also sensitive to pre- and peri-natal neurotoxic effects of psychoactive drugs [56,57].

Our results show alterations in selective features of pup vocalizations which parallel some of those currently adopted to analyze infant cry, including number and duration of calls (utterance), peak frequency (hyperphonation), and latency to emit the first call. These results thus candidate rodent neonatal USVs as an early marker in preclinical studies and potential predictors of CPF long-term effects on emotional and social competencies already reported at adulthood [10-12]. In addition, the present results support the use of acoustic cry analysis in the screening for the effects of developmental exposure to OP pesticides in humans. Selected cry features have been associated with condition of potential neurological insults such as prematurity [58], developmental exposure to metals [59], and substance of abuse such as alcohol and cocaine [60,61]. More importantly, in human infants atypical cry has been also reported concomitantly with poor performance on Brazelton-modified scale [61], a neurological assessment methodology analogues to the neurological reflex battery also applied to mouse pups in the present study [29].

Although we can not exclude that alterations in ultrasonic vocalization resulting from CPF exposure are also present later on in development, our results show a peculiar age profile with a significant decrease only on PND 10 and absence of effects on PND 4 and 7; in other words the impact of the prenatal CPF exposure was detectable only during the second postnatal week and not before, i.e. closer to time of exposure. We can only speculate on the biological mechanisms underlying the peculiar time course of prenatal CPF effects on USVs. Neonatal USV response is modulated by multiple neurotransmitter systems, and both the cholinergic [35,62] and the serotonergic circuitries have been involved in USVs. Both these two neurotransmitter systems are considered primary targets of developmental CPF exposure at doses not involving significant inhibition of AChE activity [1,63,64]. However, cholinergic and serotonergic manipulations differently affect the USV profile, with effects detectable at all neonatal ages considered, as early as PND 3 for serotonergic [65] and PND 5 for cholinergic agents [35].

USVs emission in neonatal rodents is also modulated by the hypothalamic neuropeptides oxytocin (OT) and vasopressin (AVP), as demonstrated by exogenous administration of the neuropeptides and their antagonists in neonatal rat pups [40,66], and by USVs recorded in OT and AVP 1b receptor knockout (V1b ko) mice [41,43].

Two lines of evidence candidate these hypothalamic neuropeptides as potential neural targets for the observed CPF effects on USVs in 10-days old animals. Firstly, we have recently shown that the same dose of prenatal CPF used in the present study induces long-term enhancement of OT protein levels in the hypothalamus, and an associated decrease of AVP expression in the same brain area, with males presenting the most intense effect [39]. Secondly, data concerning the profile of OT receptor during the early phases of postnatal development clearly indicate a peak of expression starting from the second postnatal week in different brain regions [42], a profile also confirmed by recent behavioral data concerning OT modulation of huddling response in 10-day-old but not in 7-day-old rats [67]. Further research on neuropeptidergic CNS levels in pups developmentally exposed to CPF is needed to assess if the changes in OT and AVP observed in adults exposed prenatally to CPF are already detectable in the first two weeks of postnatal life and are then associated with USV changes, or if such deficit in vocalizations is a component of a general delay in motor activation as signaled by the alteration in spontaneous motor behavior.

Finally, our data suggest for the first time behavioral changes in females administered with CPF during pregnancy, in the absence of overt toxicity signs. CPF did not alter the time to retrieve the male pup, but increased the level of exploratory activity (Wall Rearing). Such activation may be a delayed effect of the transient inhibition of brain AChE, even if at the time of testing four days had elapsed from the last CPF administration, and AChE brain activity levels were back to control levels (see [10]). CPF tended to increase licking after the reunion with the male pup, but this effect was likely due to a more active response to novelty, rather than to alteration of specific aspects of maternal care. Alterations in pups' licking has been evidenced in females developmentally exposed to CPF, in the absence of significant effects on general maternal responsiveness [10,12]. A more detailed analysis of how CPF interferes with the unique metabolic and endocrine condition represented by pregnancy and with the establishment of the mother-offspring bond appears warranted.

## Conclusion

Overall, results from this study show that a prenatal CPF exposure restricted to 4 days in late gestation is sufficient to determine a poor behavioral outcome characterized by diminished responsiveness to a distress condition and reduced motor activity. The specificity of the behavioral responses affected by CPF in the present study confirms that developmental CPF targets, among the others, behavioral domains involved in the regulation of affective states, a feature previously reported both in rat and mouse studies at adulthood. A further important aspect is the *translational *value of our results, as the changes in USV patterns in rodents suggest to clinicians to consider the pattern of infant cry, further to maturation of sensorimotor reflexes, as early marker for the evaluation of the risk associate to developmental OP exposure.

## Abbreviations

AChE: Acetylcholinesterase; ANOVA: analysis of variance; CNS: Central Nervous System; CPF: Chlorpyrifos; DAP: dialkylphosphate; EPA: Environmental Protection Agency; GD: gestational day; hr: hour; OPs: organophosphates; OT: oxytocin; PNDs: postnatal days; USVs: ultrasound vocalizations; US: United States; Veh: vehicle; AVP: Vasopressin.

## Competing interests

The authors declare that they have no competing interests.

## Authors' contributions

AV and LR contributed to the collection and analysis of data. AV, LR, and GC conceived and designed the study and wrote the paper. MLS contributed to revise critically the draft, especially on what concerns discussion of USV results. All authors read and approved the final manuscript.
